# The diagnostic value of the Short Physical Performance Battery for sarcopenia

**DOI:** 10.1186/s12877-020-01642-4

**Published:** 2020-07-13

**Authors:** Steven Phu, Ben Kirk, Ebrahim Bani Hassan, Sara Vogrin, Jesse Zanker, Solange Bernardo, Gustavo Duque

**Affiliations:** 1 Australian Institute for Musculoskeletal Science (AIMSS), The University of Melbourne and Western Health, Sunshine Hospital, 176 Furlong Road, St Albans, VIC 3021 Australia; 2 Department of Medicine-Western Health, The University of Melbourne, 176 Furlong Road, St. Albans, 3021 VIC Australia; 3 Subacute and Aged Care Services, Western Health, St. Albans, VIC Australia

**Keywords:** Sarcopenia, Diagnosis, Short physical performance battery, Ageing

## Abstract

**Background:**

Sarcopenia is defined as the age-related loss of muscle mass, strength, and physical performance. The original European Working Group on Sarcopenia in Older Persons (EWGSOP1) definition, and its revision (EWGSOP2), provide new cut-points and alternate measures for sarcopenia diagnosis. However, sarcopenia is rarely diagnosed in clinical settings owing to its labor-intensive diagnostic process. Given the Short Physical Performance Battery (SPPB) is a quick, easily administrable, and objective measure of muscle strength and physical performance, both of which are key components of sarcopenia, this study examined the diagnostic value of the SPPB for this muscle disease.

**Methods:**

A cross-sectional analysis of 294 community-dwelling older persons (≥65 years) was conducted. Appendicular lean body mass [(ALM) divided by height squared (ALM/h^2^)], muscle strength (handgrip/sit to stand), and physical performance [gait speed, timed up and go (TUG) and SPPB] were assessed using validated procedures, while participants were diagnosed with sarcopenia following the EWGSOP1 and EWGSOP2 criteria. Diagnostic ability of the SPPB independently and combined with ALM/h^2^ for sarcopenia was determined using area under the curve (AUC). Potential cut-points were identified, and sensitivity and specificity calculated.

**Results:**

Prevalence of sarcopenia ranged from 4 to 16% depending on the definition. The SPPB demonstrated moderate (AUC = 0.644–0.770) value in diagnosing sarcopenia, and a cut-point of ≤8points in SPPB performance resulted in high sensitivity (82–100%) but low specificity (36–41%) for diagnosing those with severe sarcopenia.

**Conclusions:**

The SPPB displayed acceptable value in diagnosing older adults with severe sarcopenia. Moreover, the high sensitivity of the SPPB when using the cut-point of ≤8 suggests it may be a favorable screening tool for sarcopenia in clinical settings where ALM measurements are not available.

## Background

Sarcopenia, the progressive loss of muscle mass, strength and function, associates with falls, fractures, disability and mortality [1]. As a result of these adverse outcomes, sarcopenia was added to the International Classification of Diseases (ICD-10), allowing for its diagnosis and management in clinical practice [2]. Currently, several definitions exist for the diagnosis of sarcopenia, with each presenting their own diagnostic criteria. These include the European Working Group on Sarcopenia in Older People (EWGSOP1 and 2), [3, 4] Sarcopenia Definition and Outcomes Consortium (SDOC), [5] International Working Group on Sarcopenia (IWGS) [6] and the population specific Asian Working Group on Sarcopenia (AWGS) [7]. In regards to the EWGSOP definition, its most recent update (EWGSOP2) [4] proposed new cut-points and a screening process beginning with the use of the SARC-F [8] for case finding. With these new cut-points and alternate assessments, wide variations in prevalence from 5 to 26% have been reported, [9] which hinders the diagnosis and management of older adults at risk.

Diagnosing sarcopenia involves assessment of muscle mass, strength, and/or physical performance. In the EWGSOP definitions, dual x-ray absorptiometry (DXA) is recommended for the assessment of appendicular lean mass (ALM), corrected for height squared (h^2^). Meanwhile, handgrip strength and gait speed are favored for assessment of muscle strength and physical performance, respectively. However, substitute measures are also proposed including the sit to stand test (alternate for handgrip strength), the timed up and go (TUG) test, and the Short Physical Performance Battery (SPPB) as alternatives for gait speed.

Of these measures, the SPPB assesses muscle strength, balance and mobility via five timed components, all of which are quick, easy to administer, and don’t require any specialized equipment [10]. As such, we sought to examine the diagnostic value of the SPPB for sarcopenia. This was carried out by first identifying the prevalence of sarcopenia using EWGSOP1 and EWGSOP2 definitions. Upon doing so, the diagnostic value of the SPPB in characterizing individuals with sarcopenia will be evaluated. Finally, cut-points for sarcopenia using the SPPB score will be identified and compared to those currently recommended.

## Methods

This was a cross-sectional study of 294 community-dwelling older adults based in Melbourne, Australia. Participants attending the falls and fractures clinic for clinical assessment between 2016 and 19 were included. Participants fulfilled the following criteria: aged over 65 years with a history of falls or at risk of falls, ability to mobilize independently with or without the use of gait aids, and no history of cognitive impairment. This study was approved by the Western Health local Human Ethics Research Committee (DB2017.13 and QA2018.80–46,205).

### Assessments

Participants undertook a comprehensive assessment which included the identification of risk for falls and fractures. Falls (in the past year) and fracture (in the past 5 years) history were determined by interview. Assessments of physical performance and balance, as well as body composition to determine ALM/h^2^ using DXA (Hologic Inc., Bedford, MA, USA) were also performed. Participant reported fear of falling was determined using the Falls Efficacy Scale – International [11].

Physical performance measures conducted on participants included the assessment of height and weight for calculation of body mass index (BMI), handgrip strength, gait speed, TUG, and SPPB. Handgrip strength was assessed using a Jamar hydraulic dynamometer (Sammons Preston Inc.) with the participant seated with their arm resting on chair arms. Participants were instructed to squeeze the dynamometer at their maximal effort, with the test performed 3 times on each side and 30 s rest provided between each trial. The best score out of the 3 trials was recorded.

To assess gait speed, we used the GAIT Rite® (CIR Systems Inc., Havertown, PA) instrumented walkway system (580 cm × 89 cm × 0.625 cm, sample rate = 120 Hz). The GAIT Rite mat was used to record spatiotemporal data. It was positioned along a straight section of the walkway and participants were instructed to walk at their normal speed. Three trials were provided with the best gait speed recorded. Gait aids were used a necessary.

To assess TUG performance, we used a 3-m course [12]. Participants began in a seated position and were instructed to stand, walk 3 m to a marked area, then return to the starting seated position. The TUG test was performed twice at the participants normal speed with the best time of completion recorded. A gait aid was allowed for use as needed.

The SPPB is an assessment which includes 5 tests for lower limb function, including balance, strength, and mobility [10]. Balance assessments were composed of 3 parts which progressed in difficulty (feet together stand, semi tandem and full tandem stand). The aim of the balance tests was to stand for 10 s unaided, with the test progressing in difficulty after successful completion. Gait speed was assessed as previously described (using GAIT Rite assessment results). The 5 times sit to stand test was performed with the participant starting in the seated position. After confirming ability to perform 1 sit to stand action, participants were then instructed to stand and sit 5 times as quickly as possible, ensuring feet were flat on the floor. Scores were allocated according to performance, with an overall maximum score of 12.

### Classifying sarcopenia

Sarcopenia status was classified based on the EWGSOP1 and EWGSOP2 definitions. This study focused on those who were classified as having severe sarcopenia and therefore fulfilled the 3 criteria for muscle strength, ALM/h^2^ and physical performance. Participants were classified as severely sarcopenic according to 7 different sarcopenia criteria. Using the EWGSOP1 definition, severe sarcopenia was defined as those with low ALM/h^2^, gait speed and handgrip strength. For the EWGSOP2 definition, 6 severe sarcopenia groups were created based on fulfillment of cut-points for muscle strength (handgrip strength or sit to stand test), ALM/h^2^ and physical performance (low gait speed, SPPB score or increased TUG time).

### Statistical analysis

Baseline characteristics for participants are presented as a median (IQR) or frequency (%). The diagnostic value of the SPPB was assessed by calculating the area under the receiver operating characteristic (ROC) curve separately for each sarcopenia definition. This was repeated with the addition of ALM/h^2^ to the model. Area under the ROC curve (AUC) of 0.7–0.8 was considered to be acceptable, with 0.8–0.9 excellent and > 0.9 outstanding [13]. Optimal cut-points for the SPPB in diagnosing sarcopenia were defined by 3 different methods: maximizing the product of sensitivity and specificity (Liu), [14] maximizing their sum (Youden) [15] and cut-point on the ROC curve closest to perfect sensitivity and specificity (Nearest 0,1) [16]. All analyses were performed using Stata 15.1 (StataCorp. 2017. Stata Statistical Software: Release 15. College Station, TX: StataCorp LLC).

## Results

### Baseline characteristics

A total of 294 participants (77.2% women) with a median age of 78 years (IQR 73, 83) were included in this analysis (Table 1). Sarcopenia prevalence ranged from 4 to 16% depending on the criteria employed (Table 2). EWGSOP1 and 2 definitions based on handgrip strength, ALM/h^2^ and gait speed showed similar prevalence of sarcopenia at 12 and 8%, respectively. Highest prevalence of sarcopenia was evident when using the sit to stand, ALM/h^2^ and SPPB or gait speed criteria at 16%, with the lowest prevalence observed when diagnosing sarcopenia by handgrip strength, ALM/h^2^ and TUG (4%). Number of falls, performance in SPPB and gait speed were similar between all sarcopenia categories except those diagnosed using TUG for physical performance. Handgrip strength varied between groups with those using the sit to stand test to diagnose low muscle strength exhibiting the highest handgrip strength. Prevalence for each sarcopenia group and baseline characteristics of participants in each sarcopenia category can be found in Table 2.

**Table 1:** Baseline characteristics of participants (*n* = 294)

	Median (IQR)
Age (yrs)	78.2 (73, 83)
Sex (women), n (%)	227 (77.2%)
Weight (kg)	70 (59, 80.8)
Height (cm)	157 (152, 164)
Body Mass Index (kg/m^2^)	28.04 (23.92, 31.45)
ALM/h^2^	6.33 (5.72, 7.22)
Falls in past year	2 (1, 3)
Fractures	1 (1, 1)
Handgrip strength (kg)	21 (16, 28)
SPPB total score	7 (5, 9)
Timed Up and Go (s)	16.14 (11.63, 21.50)
Gait Speed (m/s)	0.70 (0.53, 0.92)
FES-I	34 (24, 45)

*ALM/h*^*2*^ Appendicular Lean Mass corrected for height squared, *SPPB* Short Physical Performance Battery, *FES-I* Falls Efficacy Scale – International

**Table 2:** Characteristics of participants according to sarcopenia groups

	EWGSOP1	EWGSOP2					
	ALM/h^2^ + HG + GS	HG + ALM/h^2^ + GS	HG + ALM/h^2^ + TUG	HG + ALM/h^2^ + SPPB	STS + ALM/h^2^ + GS	STS + ALM/h^2^ + TUG	STS + ALM/h^2^ + SPPB
Prevalence	36 (12%)	22 (8%)	12 (4%)	19 (7%)	46 (16%)	24 (8%)	47 (16%)
Age (yrs)	81.5 (76, 84)	82.5 (76, 84)	83 (78.5, 84.5)	82 (76, 85)	80 (75, 84)	80.5 (76, 84)	81 (76, 85)
Sex (women), n (%)	22 (61%)	14 (64%)	7 (58%)	12 (63%)	32 (70%)	16 (67%)	32 (68%)
Weight (kg)	57.3 (50.6, 67.6)	55.3 (50, 66)	56.7 (50.1, 65.7)	55.2 (50, 66)	57.6 (51, 64.1)	56.3 (50.2, 63.5)	56 (50, 64)
Height (cm)	155 (151, 163)	154 (149, 163)	156 (151, 163)	155 (152, 163)	156 (151, 163)	157 (150, 165)	157 (152, 163)
Body Mass Index	23.79 (21.83, 26.19)	23.41 (20.81, 26.01)	23.39 (21.44, 25.61)	22.97 (20.28, 25.20)	23.26 (21.17, 26.01)	22.49 (21.45, 24.64)	23.23 (20.81, 25.20)
ALM/h^2^	5.42 (5.12, 5.92)	5.40 (5.12, 5.67)	5.38 (5.04, 5.58)	5.26 (5.04, 5.67)	5.22 (5.00, 5.67)	5.23 (5.02, 5.58)	5.23 (5.03, 5.83)
Falls in past year	2 (1, 2)	2 (1, 2)	2 (1, 3.5)	2 (1, 3)	2 (1, 2)	2 (1, 2.5)	2 (1, 3)
Handgrip strength (kg)	16 (12, 20)	13.5 (11, 17)	13.5 (12, 19)	12 (11, 18)	20 (15, 24)	19.5 (15, 22)	20 (14, 24)
SPPB score	5.5 (4.5, 7.0)	6 (4, 8)	4 (2.5, 6.5)	5 (4, 7)	5.5 (4, 7)	4.5 (4, 6)	6 (4, 7)
Timed Up and Go (s)	19.31 (16.71, 24.39)	19.50 (16.71, 24.39)	25.09 (22.27, 33.94)	19.77 (16.81, 25.80)	19.46 (16.71, 24.39)	25.09 (21.62, 29.92)	19.23 (15.42, 24.39)
Gait Speed (m/s)	0.54 (0.39, 0.69)	0.51 (0.38, 0.66)	0.38 (0.26, 0.61)	0.50 (0.38, 0.65)	0.56 (0.40, 0.70)	0.46 (0.33, 0.62)	0.56 (0.40, 0.71)
FES-I	36 (25.5, 48.5)	36.5 (29, 52)	50 (29, 59.5)	38 (29, 57)	37 (26, 48)	43.5 (26.5, 52.5)	36 (25, 48)

*ALM/h*^*2*^ Appendicular Lean Mass corrected for height squared, *GS* Gait speed, *HG* Handgrip strength, *SPPB* Short Physical Performance Battery, *TUG* Timed up and go, *FES-I* Falls Efficacy Scale – International

### Diagnostic value of SPPB

Use of the SPPB resulted in moderate diagnostic value for sarcopenia for all EWGSOP definitions with AUC ranging from 0.644 to 0.770, as shown in Table 3. The lowest AUC occurred when using the SPPB to diagnose sarcopenia by the recommended EWGSOP2 criteria of low handgrip strength, ALM/h^2^ and gait speed (AUC = 0.644). Highest AUC for use of the SPPB was found when diagnosing EWGSOP2 sarcopenia according to the alternate measures of sit to stand, ALM/h^2^ and TUG (AUC = 0.770).

**Table 3:** Area under curve (AUC) for use of Short Physical Performance Battery (SPPB) alone, or in combination with Appendicular Lean Mass corrected for height (ALM/h^2^)

Definition	Criteria	AUC SPPB	AUC SPPB + ALM/h^2^
EWGSOP1	ALM/h^2^ + GS + HG	0.678	0.846
EWGSOP2	HG + ALM/h^2^ + GS	0.644	0.849
	HG + ALM/h^2^ + TUG	0.767	0.870
	HG + ALM/h^2^ + SPPB	0.715	0.873
	STS + ALM/h^2^ + GS	0.701	0.893
	STS + ALM/h^2^ + TUG	0.770	0.897
	STS + ALM/h^2^ + SPPB	0.703	0.889

*GS* Gait speed, *HG* Handgrip strength, *SPPB* Short Physical Performance Battery, *TUG* Timed up and go

### Diagnostic value of SPPB combined with ALM/h^2^

When combining the SPPB with ALM/h^2^, there was a significant increase in the diagnostic capability. AUC for both the EWGSOP1 and 2 was increased, with AUC in an ‘excellent’ range for all groups, between 0.846 and 0.897. The lowest AUC was evident using the EWGSOP1 criteria for severe sarcopenia (AUC = 0.846), with AUC observed in the EWGSOP2 criteria of sit to stand, ALM/h^2^ and TUG performance (AUC = 0.897). An overview of the AUC for use of the SPPB combined with ALM/h^2^ in diagnosing each severe sarcopenia group can be found in Table 3.

### Potential SPPB cut-points for diagnosing sarcopenia

Optimal cut-points for use of the SPPB alone to diagnose sarcopenia varied depending on method and ranged from 4 to 8 points. All cut-points resulted in either high sensitivity and low specificity or vice versa (Table 4). The currently recommended cut-point of ≤8 produced high sensitivity (82–100%) but low specificity (36–41%) for all sarcopenia definitions. Most commonly observed optimal cut-points in SPPB performance were 5 and 6, which resulted in sensitivity and specificity between 60 and 75%.

**Table 4:** Sensitivity and specificity for cut-points for sarcopenia based on definition

Definition	Criteria	Method	Cut-point (≤)	Sensitivity (95% CI)	Specificity (95% CI)
EWGSOP1	ALM/h^2^ + GS + HG	Liu, Youden	7	81% (64, 92)	48% (41, 54)
		Nearest (0,1)	6	61% (44, 77)	62% (55, 68)
		Current literature	8	89% (74, 97)	38% (32, 44)
EWGSOP2	HG + ALM/h^2^ + GS	Liu, Youden	5	46% (24, 68)	74% (68, 79)
		Nearest (0,1)	6	55% (32, 76)	60% (54, 66)
		Current literature	8	82% (60, 95)	36% (30, 42)
	HG + ALM/h^2^ + TUG	Liu, Nearest (0,1)	5	67% (35, 90)	74% (68, 79)
		Youden	4	58% (28, 85)	84% (79, 88)
		Current literature	8	92% (62, 100)	36% (30, 41)
	HG + ALM/h^2^ + SPPB	Liu,	7	84% (60, 97)	46% (40, 52)
		Youden	8	100% (82, 100)	37% (31, 43)
		Nearest (0,1)	6	63% (38, 84)	60% (54, 66)
		Current literature	8	100% (82, 100)	37% (31, 43)
	STS + ALM/h^2^ + GS	Liu, Nearest (0,1)	6	65% (50, 79)	63% (57, 69)
		Youden	8	94% (82, 99)	40% (33, 46)
		Current literature	8	94% (82, 99)	40% (33, 46)
	STS + ALM/h^2^ + TUG	All	5	67% (45, 84)	76% (70, 81)
		Current literature	8	96% (79, 100)	37% (31, 43)
	STS + ALM/h^2^ + SPPB	Liu, Youden	8	100% (93, 100)	41% (35, 47)
		Nearest (0,1)	6	64% (49, 77)	63% (57, 69)
		Current literature	8	100% (93, 100)	41% (35, 47)

*ALM/h*^*2*^ Appendicular Lean Mass corrected for height, *GS* Gait speed, *HG* Handgrip strength, *SPPB* Short Physical Performance Battery, *TUG* Timed up and go

## Discussion

We examined the diagnostic value of the SPPB alone and in combination with ALM/h^2^ in diagnosing sarcopenia in community-dwelling older adults. We characterized the sensitivity and specificity of currently recommended cut-points, in addition to new cut-points based on our study population, and found two main findings. Firstly, the SPPB alone resulted in moderate diagnostic value but increased to an excellent level when combined with DXA measured ALM/h^2^. This was regardless of which definition (EWGSOP1 or EWGSOP2) was employed. Secondly, the currently recommended SPPB cut-point of ≤8 showed high sensitivity for sarcopenia, however, a more conservative cut-point of ≤6 provided the greatest specificity. It therefore appears that the SPPB represents an objective screening tool to diagnose those at risk of sarcopenia.

The SPPB has been used to monitor physical performance in both healthy community-dwelling older adults as well as hospitalized patients [17–20]. This measure is advantageous in that minimal equipment is required to perform the test, and objective data is provided on muscle strength, gait speed and standing balance. In addition, specific components of the SPPB can independently predict the risk of declines in activities of daily living, [21] falls, [22] hospitalization [23] and mortality [23]. Furthermore, the SPPB can be used as a sensitive monitoring tool for lower limb physical performance with a change of 1 point considered clinical significant [24]. These factors place the SPPB as an ideal tool for the diagnosis of sarcopenia in clinical settings and subsequent monitoring of resistance exercise (RE) interventions which are, at present, the most efficacious treatment for this muscle disease [25]. While other tools such as handgrip strength are reliable, easy to administer and a good marker of whole-body strength, they are poor responders to RE interventions [26]. Thus, the strength component of the SPPB should be assessed by sit to stand time, which is a reliable marker of lower limb strength and is sensitive to change [4].

Our findings show the SPPB displayed acceptable diagnostic value for sarcopenia. When employing the most common cut-points of ≤6, sensitivity and specificity was 65 and 63%, respectively. This cut-point has been associated with more than 3-fold likelihood of recurrent falls for both men and women, [27] one of the primary adverse outcomes of sarcopenia. Indeed, those diagnosed as sarcopenic (using the EWGSOP1) reported a 3-fold increased risk of falls compared to non-sarcopenic adults [28]. When increasing this cut-point to ≤8, the sensitivity and specificity increased to 100 and 41%, respectively. In line with this, this is the cut-point recommended for poor physical performance by the EWGSOP criteria. Despite the excellent sensitivity, the poor specificity of this cut-point indicates that the SPPB cannot be solely used for the diagnosis of sarcopenia.

Highly sensitive tests have been proposed as ideal tools for screening purposes, allowing for the early identification and initiation of treatment [29]. Meanwhile, high specificity has been reported as the key diagnostic factor for a disease, with a combination of high sensitivity/low specificity and low sensitivity/high specificity test an ideal method to address false positives [29]. Using this model, the SPPB displays high sensitivity and represents the ideal screening tool for sarcopenia. Alternately, the SARC-F tool has consistently shown high specificity in numerous studies [30, 31] and may therefore facilitate the diagnostic process. This combined approach represents an area for further research given the potential to enhance clinical care.

Sarcopenia in its current form includes the assessment of muscle strength, mass, and physical performance. However, whilst measures of strength and physical performance have shown links to adverse outcomes of sarcopenia, the same relationship is not observed with ALM [32, 33]. Our findings corroborate previous studies which have shown the disconnect between measures of muscle strength and physical performance and muscle mass. This is represented by the poor to acceptable AUC when using the SPPB as an independent diagnostic tool. However, a combination of SPPB with ALM/h^2^ resulted in increased AUC, matching well with both initial and revised EWGSOP sarcopenia criteria, with the highest diagnostic value evident using the sit to stand test combined with ALM/h^2^ and either gait speed or SPPB. This was somewhat expected given both the sit to stand test and gait speed are already components of the SPPB. However, it was surprising to observe that the combination of SPPB and ALM/h^2^ yielded an increased AUC for all the other diagnostic criteria proposed by EWGSOP2. Given the SPPB is a tool which can be easily implemented into the routine care of patients without a need for specialized equipment, this finding may provide useful in clinical practice.

In the current study, we diagnosed participants presenting with severe sarcopenia which is a strength given that our previous findings showed that the severity of sarcopenia was the driving factor that increased falls and fractures [34]. Studies assessing the impact of sarcopenia in modulating balance performance are required to further efforts in developing interventions. The findings of the present study should also be taken in context of the limitations. Firstly, our population, although community-dwelling, reported a history of falls and/or fractures. Therefore, results may have been biased given that these participants are at increased risk for sarcopenia. Secondly, the cross-sectional nature of our study limits our findings as we are unable to determine whether longitudinally the participants classified as sarcopenic experienced adverse outcomes such as falls. Employing a longitudinal study design would have strengthened the findings.

## Conclusion

This study provides an insight into the use of the SPPB for diagnosing sarcopenia according to the initial and revised EWGSOP definitions. Despite the acceptable diagnostic value, no cut-points could be determined which provided high sensitivity and specificity. However, a cut-point of ≤8 in the SPPB showed high sensitivity. Therefore, the SPPB may be used as a screening tool for sarcopenia to trigger further investigations and early intervention. The combination of the highly sensitive SPPB with a highly specific test may prove to be of great use to clinicians for diagnosing and treating sarcopenia, however, requires further investigation in longitudinal trials.

## Data Availability

The datasets used and/or analyzed during the current study are available from the corresponding author on reasonable request.

## Abbreviations

EWGSOP: European Working Group on Sarcopenia in Older People
SPPB: Short Physical Performance Battery
DXA: Dual X-ray Absorptiometry
ALM/h^2^: Appendicular Lean Mass corrected for Height squared
TUG: Timed Up and Go
AUC: Area Under receiver operating Curve
